# New Strategies to Overcome Resistance to Chemotherapy and Immune System in Cancer

**DOI:** 10.3390/ijms20194783

**Published:** 2019-09-26

**Authors:** Chiara Riganti, Marialessandra Contino

**Affiliations:** 1 Department of Oncology, Oncological Pharmacology Lab, University of Torino via Santena 5/bis, 10126 Torino, Italy; 2 Department of Pharmacy, University of Bari ALDO MORO, via Orabona 4, 70125 Bari, Italy

**Keywords:** multidrug resistance (MDR), chemotherapy, immunotherapy, cancer stem-like cells

MultiDrug resistance (MDR) is a complex phenomenon responsible for multiple cross-resistance towards structurally unrelated drugs and it characterizes almost 70% of solid and haematological tumours at the diagnosis. This percentage further increases after treatments, because chemotherapy selects and expands resistant clones. MDR is often related to the expression of drug efflux transporters belonging to the ATP Binding Cassette (ABC) family, that pump drugs outside the cells limiting their intracellular accumulation and toxicity. However, ABC transporters are not the only cause of resistance. Notably, MDR cells are very often simultaneously resistant to chemotherapy, radiotherapy, hypoxia, and nutrient shortages. In addition, MDR cells are also characterized by a high immune-evasive capacity, since they have poor immune-activating molecules on their surface and/or produce immune-suppressive metabolites that do not allow to the host immune system to mount an anti-tumour response. The reasons of this chemo-immune-resistance of MDR cells is due to their ability to adapt to stressors. The chemo-immune-resistant phenotype can be considered part of a multi-stress resistant phenotype.

This Special Issue reports different mechanisms involved in chemo-immune-resistance and different strategies that have been employed to overcome MDR, e.g., (i) by restoring oxidative stress sensitivity of MDR cells rehabilitating the cytotoxicity of specific antineoplastic drugs in different type of cancers; (ii) by identifying new targets to rehabilitate the response to chemo- and immuno-therapies. Among the different mechanisms recently identified, a rewiring of cell metabolism and consequent intracellular pH plays critical role. For instance, constitutively chemo-resistant mesothelioma and colon cancer cells with acquired drug resistance differ from chemo-sensitive counterparts for a lower metabolic flux though glycolysis and oxidative phosphorylation metabolism. Surprisingly, the addition of pyruvate reverses the resistance to doxorubicin, opening the possibility to potential metabolic modifiers as chemosensitizers [1]. The higher glycolytic flux often implies an altered intracellular pH, thereby increasing the activity of pH-dependent intracellular organelles such as autophagic-lysosomes that mediate a cytoprotective autophagy. Exploiting this feature, Zhan and colleagues successfully employed a new autophagy inhibitor, alpha-hederin, which alters lysosomal pH and inhibits the lysosomal maturation of cathepsin D. The burden of undigested materials leads to the accumulation of reactive oxygen species (ROS). These mechanisms make alpha-hederin an effective agent in reversing the resistance to paclitaxel in resistant non-small cell lung cancer (NSCLC) [2]. 

Since chemoresistance is also caused by genetic mutations affecting different proteins involved in the cell cycle, apoptosis and cell adhesion, targeting these mechanisms could be strategical in reversing MDR. Indeed, combined alterations in the cell cycle, apoptosis and cell adhesion all contribute to chemoresistance. Only combination treatments targeting these different pathways may result a winning strategy in overcoming MDR [3]. The constant search of novel mechanisms involved in cell death or proliferation has led to the identification of interesting and unexpected targets that can be modulated to overcome MDR. For instance, the over-expression of the anti-apoptotic protein Bcl-2 has been proposed as one of cancer hallmarks and its inhibition is a promising strategy to face cancer. Bcl-2 plays a pivotal role in apoptosis resistance to anticancer agents. Therein Bcl-2 inhibitors may become of great utility in overcoming resistance to chemo- and immune-therapy [4]. Caspase-8, originally identified as a pivotal pro-apoptotic protein down-regulated in several tumours, shows a different behaviour in glioblastoma, where its high levels correlate with a worse prognosis. In glioblastoma, caspase-8 induces NF-κB-dependent expression of several pro-inflammatory cytokines, angiogenesis, and in vitro and in vivo tumorigenesis, suggesting that in this tumour it loses its pro-apoptotic activity gaining other functions related to tumour aggressiveness and resistance to therapy. Therefore inhibitors of caspase-8 may be regarded as new adjuvant agents in chemo-refractory glioblastoma [5]. Besides increasing survival and decreasing apoptosis, also enhancing detoxification reactions is important to resist to stressors as chemotherapy. Glutathione transferases (GSTs) are a superfamily of phase II enzymes, overexpressed in different type of cancers. Although each cancer type has a unique GST signature, overall GST overexpression has been clearly linked with the onset of chemo-resistance. Using specific GST inhibitors and/or pro-drugs, derivatives of conventional anti-cancer drugs and selectively targeting GST-overexpressing cancers more prone to developing chemoresistance, is a new strategy overcoming MDR [6]. If several proteins induce MDR, then other potential chemo-sensitizing factors have been identified. Tripartite motif (TRIM) proteins modulate p53 degradation and tips the balance between a cytotoxic and a cytoprotective reaction towards the damages induced by therapy. For this reason, TRIM proteins have recently emerged as chemosensitizers in different types of cancers [7].

Another feature that often induces the MDR phenotype is the presence of cancer stem-like cells (CSCs) that are resistant to most anti-cancer treatments. One reason is the high expression of multiple ABC transporters in CSCs. Glioblastoma, for instance, the most common primary malignant tumour of the central nervous system, displays poor prognosis with a resounding resilience against all current treatments, mainly because of the presence of glioblastoma stem-like cells (GSCs). GSCs are rich of the multidrug resistance-associated protein 1 (MRP1) transporter, determining the efflux of several anti-glioblastoma drugs and poor prognosis. Recently, FK506 (Tacrolimus) has been found to chemo-sensitize GSCs to MRP1 substrates, thus representing a potential chemosensitizer treatment specifically targeting the GSCs component [8]. Different strategies are nowadays employed to target CSCs, such as gold nanoparticles that have emerged as innovative tools for photo-thermal therapy. In particular, functionalized gold nanoparticles represent a significant advance in the treatment of chemo-resistant metastatic cancers [9]. Since glioblastoma therapy is multi-modal, not only resistance to chemotherapy but also to radiotherapy, dramatically impacts the prognosis. We have a still poor understanding of glioblastoma biology and of the chemo-radio-resistance acquisition in this tumour. Among the novel biomarkers predictive of resistance, acid ceramidase was one of the latest factors identified in the development of radio-resistance [10]. 

Besides glioblastoma, two other cancers—pancreatic ductal adenocarcinoma (PDAC) and triple-negative breast cancer (TNBC)—are particularly chemo-resistant, and therefore are the object of intensive studies. PDAC is often diagnosed at an unresectable stage due to metastases or local extension. Given the low rate of success of chemotherapy, immune-therapy—based on the anti-CTLA-4 antibody ipilimumab and the anti-PD-1 antibodies pembrolizumab and nivolumab—has been approved. However, immune-therapy results are not satisfactory in PDAC. This low success is due to complex crosstalk between the tumour microenvironment, immune cells, and tumour cells that promote PDAC development. Disrupting such crosstalk, i.e., by preventing the activation of pro-inflammatory pathways, can be regarded as a new strategy to overcome immune resistance in this tumour [11]. TNBC is one of the most aggressive types of breast cancer, because of the absence of estrogen receptor (ER), progesterone receptor (PR), and human epidermal growth factor receptor 2 (HER2) limits the possibility of using hormone-therapy or anti-Her2 targeted therapy. Chemotherapy is the main therapeutic option, but it is less effective than in other cancer type because of the intrinsic resistance of TNBC. The analysis of differentially expressed genes and their biological roles in different TNBC drug-resistant cell lines allowed to identify several pathways activated by cytokines responsible for chemoresistance, opening a new spectrum of therapeutic opportunities [12].

Overall, the bases of the chemo-immune-resistance of cancer cells likely rely on the superior ability of MDR cells to adapt to stressing conditions. Several molecular circuitries and tumour microenvironment-dependent factors—not fully understood—simultaneously determine such resistance. In this Special Issue, we provided insights on the latest and unexpected biomarkers and pathways predictive of chemo-immune-resistance and exploitable as potential targets to achieve chemo-immune-sensitization in the most refractory tumours. 
